# An arm swing enhances the proximal-to-distal delay in joint extension during a countermovement jump

**DOI:** 10.1038/s41598-024-70194-z

**Published:** 2024-09-02

**Authors:** Christina M. Cefai, Joseph W. Shaw, Emily J. Cushion, Daniel J. Cleather

**Affiliations:** 1 Faculty of Sport, Technology and Health Sciences, St Mary’s University, Twickenham, UK; 2 Ballet Healthcare, The Royal Ballet, Royal Opera House, London, UK; 3 School of Sport, Rehabilitation and Exercise Sciences, University of Essex, Colchester, UK; 4 Institute for Globally Distributed Open Research and Education (IGDORE), London, UK

**Keywords:** Principal component analysis, Motor control strategy, Mechanical coupling, Degrees of freedom, Proximal-to-distal, Arm swing, Musculoskeletal system, Biomedical engineering

## Abstract

An abundance of degrees of freedom (DOF) exist when executing a countermovement jump (CMJ). This research aims to simplify the understanding of this complex system by comparing jump performance and independent functional DOF (fDOF) present in CMJs without (CMJ_NoArms_) and with (CMJ_Arms_) an arm swing. Principal component analysis was used on 39 muscle forces and 15 3-dimensional joint contact forces obtained from kinematic and kinetic data, analyzed in FreeBody (a segment-based musculoskeletal model). Jump performance was greater in CMJ_Arms_ with the increased ground contact time resulting in higher external (*p* = 0.012), hip (*p* < 0.001) and ankle (*p* = 0.009) vertical impulses, and slower hip extension enhancing the proximal-to-distal joint extension strategy. This allowed the hip muscles to generate higher forces and greater time-normalized hip vertical impulse (*p* = 0.006). Three fDOF were found for the muscle forces and 3-dimensional joint contact forces during CMJ_NoArms_, while four fDOF were present for CMJ_Arms_. This suggests that the underlying anatomy provides mechanical constraints during a CMJ, reducing the demand on the control system. The additional fDOF present in CMJ_Arms_ suggests that the arms are not mechanically coupled with the lower extremity, resulting in additional variation within individual motor strategies.

## Introduction

In human movement, the movement of multiple segments, each with six degrees of freedom (DOF), is coordinated through motor control strategies^1^. The movement (or resistance to movement) of these segments is created by the muscles acting upon each segment, where many more muscle activation strategies exist than segmental kinematic DOFs. This results in many possible strategies to achieve the same outcome, and therefore an abundance of possible motor control strategies, allowing an individual to adapt to additional or unexpected tasks and demands^2^. This creates an indeterminant problem with more DOFs present than constraints^1^.

Researchers have shown that the number of DOFs utilized by individuals can be reduced, for example, through conscious freezing of DOF during motor skill learning^3–6^. Neuromechanical synergies^7^, mechanical coupling,^8,9^ and the anatomy of the musculoskeletal system itself^10^ effectively create additional constraints on the system which can reduce the number of independent DOF and the demand on the central control system^8^. The reduced number of independent DOF results in fewer motor control strategies that are explored to achieve the same outcome^7^. While the constraints imposed by the system’s anatomy do not change between tasks, the demands and complexity of a task can change the number of independent DOF observed. Increased loading has shown to reduce the independent DOF and therefore results in less variation exhibited by the chosen movement strategies^7^. However, increased complexity of a task has shown to increase the number of independent DOF, with more motor control strategies being explored to achieve an outcome, thus increasing the demand on the central control system^11^. One of the methods used to simplify the understanding of this complex motor control system is by identifying what have been termed “functional degrees of freedom” (fDOF), representing the main characteristics of the movement system^1^. These are found through the dimensional reduction technique of principal component analysis (PCA), transforming the original data into a new orthogonal coordinate system, and reducing the correlation that may be present between DOF^12^. Therefore, the fDOF are defined by the minimum number of independent principal components (PCs) required to define a high percentage of variance in the original data^1^.

During the countermovement vertical jump (CMJ), a common movement pattern observed is the proximal-to-distal strategy, where proximal segments begin to rotate before their distal segment^13^. This improves the mechanical efficiency of the movement and increases jump height, compared to the simultaneous acceleration of all segments^14^. Adding an arm swing to a CMJ improves jump performance through a combination of factors. Firstly, the ground reaction force (GRF) profile is altered^15^ and the time of the countermovement is prolonged, increasing the net impulse and, therefore, take-off velocity^16–19^. Secondly, a delay in the proximal-to-distal strategy is observed to allow the arms to accelerate upwards before extending the lower limbs^17,20^, resulting in increased net joint moments (NJM) at the hip and ankle^16–18,20,21^. Thirdly, the arm swing itself creates a ‘pull mechanism’ in which the shoulder flexor muscles increase the vertical work done and energy generated^15,18,22^ and increases the height of the center of mass at take-off^16,18^.

While research has looked into the effects of an arm swing on jump performance, its effect on independent DOF present in muscular activation and 3-dimensional (3D) joint contact forces (JCF) is currently unknown. It is hypothesized that adding an arm swing will increase the variation observed in muscle forces and 3D JCFs at an individual participant and group level, increasing the number of fDOF present for the CMJ. Therefore, the purpose of this study is two-fold. Firstly, external and lower-limb joint impulses will be compared in a CMJ with (CMJ_Arms_) and without an arm swing (CMJ_NoArms_) to assess the impact of arm swing on jump performance for the participants within this study. The second aim is to compare the fDOF exhibited in CMJ_NoArms_ and CMJ_Arms_ for the 3D JCF and muscle forces.

## Methods

### Study design and participants

A cross-sectional study design was utilized to investigate the effect of an arm swing on a CMJ. The data used in this study were collected previously by Cushion et al.^13^. Participants included in the study had to be between the ages of 18 and 50 and currently involved in any regular sport or physical training activities. Participants were excluded if they had any musculoskeletal injuries at the time of testing. Twenty-one healthy participants (10 women: height = 167.4 ± 6.9 cm, weight = 62.9 ± 7.3 kg; 11 men: height = 178.0 ± 7.6 cm, weight = 82.4 ± 7.2 kg) volunteered to participate in this study and gave written informed consent after understanding the details of the study. Ethical approval was provided by the ethics sub-committee of St Mary’s University, Twickenham. The study was conducted in line with the guidelines in the Declaration of Helsinki.

### Procedure

Participants attended a single data collection session in which anthropometric measures of height and weight were taken and were provided with a standardized shoe according to their shoe size. Reflective markers were placed on anatomical landmarks on the participants’ pelvis (left and right anterior superior iliac spine and posterior superior iliac spines) and right lower limb, according to Cleather and Bull^23^. The lower limb markers were placed on the medial and lateral femoral epicondyle, apex of the medial and lateral malleolus, posterior aspect of the calcaneus, head of the second metatarsal and tuberosity of the fifth metatarsal. An additional tracking marker was placed on the foot, and 3 additional markers were secured to the mid-thigh and anterior tibial shaft using rigid plates. A standardized warmup consisting of bodyweight squats, lunges, inchworms, hip rotations and vertical jumps was performed by all participants. A Vicon 14-camera motion capture system (Vicon MX System, Nexux2.2 software, Vicon Motion System Ltd, Oxford, UK) was used to record kinematic data at 200 Hz, synchronously with kinetic data recorded at 1000 Hz using two force plates (Kistler Type 9287BA, BioWare 3.24 software, Kistler Instruments Ltd, Hampshire, UK).

Following the warmup, all participants were asked to perform five separate maximum effort CMJs with their hands placed on their hips for the entire trial (CMJ_NoArms_) and another five separate maximum effort CMJs with the use of an arm swing (CMJ_Arms_). For all jumps, participants were instructed to take-off and land with one foot on each force-plate. A self-selected duration of break was taken between individual jumps, and a two-minute break was given between the different jumps to mitigate fatigue. The order of jumps was counterbalanced to avoid any order effect.

### Data analysis

The kinematic and kinetic data were preprocessed using a 5th order Woltring filter with a cutoff frequency of 10 Hz^13^. The start of the movement was defined as the frame when the right anterior superior iliac spine marker began to descend below stationary height and ended when the GRFs were 0 N on take-off^13^. FreeBody^23^—an open-source segment-based musculoskeletal model of the lower limb—was used to create the participants’ scaled musculoskeletal models composed of five rigid segments with six kinematic DOF each (the foot, shank, thigh, pelvis, and patella), 163 muscle elements defining 39 muscles, and 14 ligament elements. The muscle, ligament, and 3D JCFs at the ankle, medial and lateral tibiofemoral joints, patellofemoral joint, and the hip were calculated at each time frame based on an optimization approach to inverse dynamics^24^ using FreeBody^23^. This was done using MATLAB’s constrained nonlinear programming solver (‘fmincon’), specifically the sequential quadratic programming (‘sqp’) algorithm (The MathWorks, Inc., MA, version R2023b) to solve the 193 unknowns with only 22 equations of motion. Where the optimization failed to solve within the muscle and ligament force constraints set according to the participant’s body mass, the upper bound limits were increased incrementally until the maximum force limit of the muscles and ligaments was increased by five times, where the smaller stabilizing muscles around the foot and ankle were the main limiting factor for a few frames. When a solution was still not found, the solver’s constraint tolerance was relaxed from 1 × 10^–6^ to 1 × 10^–3^. Trials were included in the data analysis only if a solution was found for the trial. This resulted in 18 participants being kept in the analysis (8 women: height = 168.1 ± 6.9 cm, weight = 62.8 ± 6.9 kg; 10 men: height = 178.0 ± 8.0 cm, weight = 82.4 ± 7.5 kg), where a solution was found for both CMJ_NoArms_ and CMJ_Arms_.

The force vectors of the 163 muscle elements were summed to define the 39 muscles and normalized to the participant’s bodyweight. The vertical external and joint impulses were calculated from the area underneath the vertical GRF and JCF-time curves respectively. The impulses were averaged across trials for each participant (participant-level impulse) before averaging across participants (group-level impulse). The modified Akima piecewise cubic Hermite interpolation (‘makima’), that is MATLAB’s specific modification of Akima’s interpolation method which reduces excessive local undulations^25,26^, was then used to time normalize all trials to 501 points in MATLAB (The MathWorks, Inc., MA, version R2023b) before recalculating the impulses from their respective force–time normalized curves, resulting in time-normalized external and joint vertical impulses.

### Statistical analysis

A Wilcoxon matched-pairs test (α = 0.05) was used to compare the participants’ external vertical impulses (not normally distributed data), with a power of 84% for a large effect size (dz = 0.8) calculated in a post hoc power analysis. A two-way repeated measure ANOVA was used to compare the vertical joint impulses, with the use of an arm swing (CMJ_NoArms_ and CMJ_Arms_) and specific joint being the two independent variables (two-way interaction: α = 0.05; main effect of the independent variables: α = 0.05). Using the Bonferroni adjustments for the 95% confidence interval and significance level (α = 0.01), and a power of 98% with a moderate effect size (f = 0.4) calculated from a post hoc analysis, a simple effects analysis was also performed to compare each joint individually between jumps. These statistical tests were repeated for the time-normalized impulses, however a repeated measures t-test was used for the time-normalized external vertical impulses as the data was normally distributed. All data were assessed for outliers beyond 1.5 times the interquartile range of the upper or lower extremity, as visualized on a box plot. It was also assessed by Shapiro–Wilk’s test of normality (α = 0.05), and by Mauchly’s test of sphericity (α = 0.05) for the five joints. The statistical analyses were conducted in IBM SPSS Statistics (The International Business Machines Corporation, NY, version 29.0.1.1) and post hoc power analyses were conducted in G*Power^27^.

The participant-level composite muscle and 3D JCF curves were calculated by averaging across each trial’s output vectors at every time point for both jumps. Group-level composite curves were also calculated by averaging the participant-level curves for each muscle. Principal component analyses were performed in MATLAB (The MathWorks, Inc., MA, version R2023b) on the participant-level muscle force and 3D JCF composite curves for CMJ_NoArms_ and CMJ_Arms_ at a participant and group level (Table 1). After assessing for the three assumptions (outliers, normality and sphericity) as defined previously, a three-way repeated measure ANOVA (three and two-way interactions and main effects of the independent variables, α = 0.05) was performed in IBM SPSS Statistics to compare the cumulative explained variation by the participant-level PCA. The cumulative explained variation was compared between the muscle and 3D JCFs (first independent variable), the use of an arm swing (CMJ_NoArms_ and CMJ_Arms_, second independent variable), and the number of PCs (PCs 1 to 3, third independent variable) at participant level. Simple effects of cumulative explained variation of the muscle and 3D JCFs at each PC level (PCs 1–3) were compared with and without the use of an arm swing and considered statistically significant at α = 0.017 (Bonferroni adjustment for 3 PC levels) with a power of 99% for a moderate effect size (f = 0.4), calculated from a post hoc power analysis using G*Power^27^. The fDOF were defined as number of PCs required to explain 95% variation of the muscle forces and 3D JCFs for CMJ_NoArms_ and CMJ_Arms_ per participant^1^.

**Table 1:** The number of PCAs performed for muscle forces and JCFs at participant and group level.

Variable	Time series data used	Analysis level	Input matrix dimensions	Number of separate analyses
Muscle forces	Participant composite muscle forces (39 muscles per participant)	Participant Level	501 data points × 39 force vectors	36 PCAs (2 jumps × 18 participants)
		Group Level	501 data points × 702 force vectors (39 muscles × 18 participants)	2 PCAs (2 jumps)
Joint contact forces	Participant composite 3-dimensional JCFs (5 joints × 3 dimensions per participant)	Participant Level	501 data points × 15 force vectors	36 PCAs (2 jumps × 18 participants)
		Group Level	501 data points × 270 force vectors (15 JCF × 18 participants)	2 PCAs (2 jumps)

Their input time series data and matrix dimensions are also defined. The analyses were performed for both jumps (CMJ_NoArms_ and CMJ_Arms_).

The 39 group-level muscle composite curves were categorized into seven muscle groups according to peak force timing and force curve profile. After listing all muscles in order of peak timings, groups 1 and 2 were separated after visual inspection of their force–time curves as group 2 had slightly later force peaks with a different profile in CMJ_Arms_. Groups 2 and 3 exhibited a large gap between muscle peak force timings in CMJ_Arms_, creating a clear distinction between groups, while muscles in group 4 had a qualitatively slower rate of force production to muscles in group 3 upon visual inspection of their force–time curves. Groups 5–7 were categorized based on their force profiles as they all exhibited a peak at or close to 100% of the countermovement time. Group 5 showed earlier activation than group 6 while group 7 muscles exhibited a unique curve profile with both a ‘bell-shape’ force curve and a peak at the end of the countermovement. The full list of grouped muscles is provided in Table 2. A linear combination of PC1 to PC5 was defined for each muscle group based on the coefficients resulting from the group-level PCAs. The muscles’ average coefficients for PC1 to PC5 were calculated from the 18 instances of the muscle within the coefficient matrix. The sum of the average coefficients for all muscles included in each group were calculated for PC1 to PC5 to define the muscle group’s PC combination. The group’s PC combination was simplified after normalizing to the highest coefficient by only retaining PC1 and PC2 with a normalized coefficient larger than 0.2 and PC3, PC4 and PC5 with a normalized coefficient larger than 0.35. When PC5 was the main contributor to the PC combination, the cutoff coefficients for PC1 to PC4 was 0.1. Different cut-off coefficients were defined due to lower PC score magnitudes found in PC3, PC4 and PC5, thus having little impact on the PC combination at lower normalized coefficients.

**Table 2:** Muscle groups used for data analysis.

Muscle group number	Muscles included
**1**	Biceps Femoris (Long Head), Obturator Internus, Quadratus Femoris, Semimembranosus, Piriformis, Gemellus
**2**	Gluteus Maximus, Vastus Medialis, Vastus Intermedius
**3**	Vastus Lateralis, Flexor Hallucis Longus, Peroneus Longus, Tibialis Posterior, Adductor Magnus, Peroneus Brevis, Flexor Digitorum Longus
**4**	Soleus, Gluteus Medius, Rectus Femoris
**5**	Gastrocnemius, Gluteus Minimus, Sartorius, Plantaris
**6**	Adductor Brevis, Adductor Longus, Biceps Femoris (Short Head), Gracilis, Iliacus, Pectineus, Psoas Major, Psoas Minor, Tibialis Anterior
**7**	Extensor Digitorum Longus, Extensor Hallucis Longus, Obturator Externus, Peroneus Tertius, Popliteus, Semitendinosus, Tensor Fascia Latae

The muscle group PC combinations (without normalized coefficients) were plotted against real time by multiplying the normalized time with the average lengths of the CMJ_NoArms_ and CMJ_Arms_ jumps. The “principal impulse” was calculated from the area under these curves by taking the minimum PC score as the base, rather than 0. The total muscle impulse for each group was calculated through the summation of the group-level average muscle impulses. A Pearson’s correlation was run in IBM SPSS Statistics to determine the relationship between “principal impulse” and muscle impulse, irrespective of jump (CMJ_Arms_ and CMJ_NoArms_).

## Results

### Jump performance

**Figure 1:**
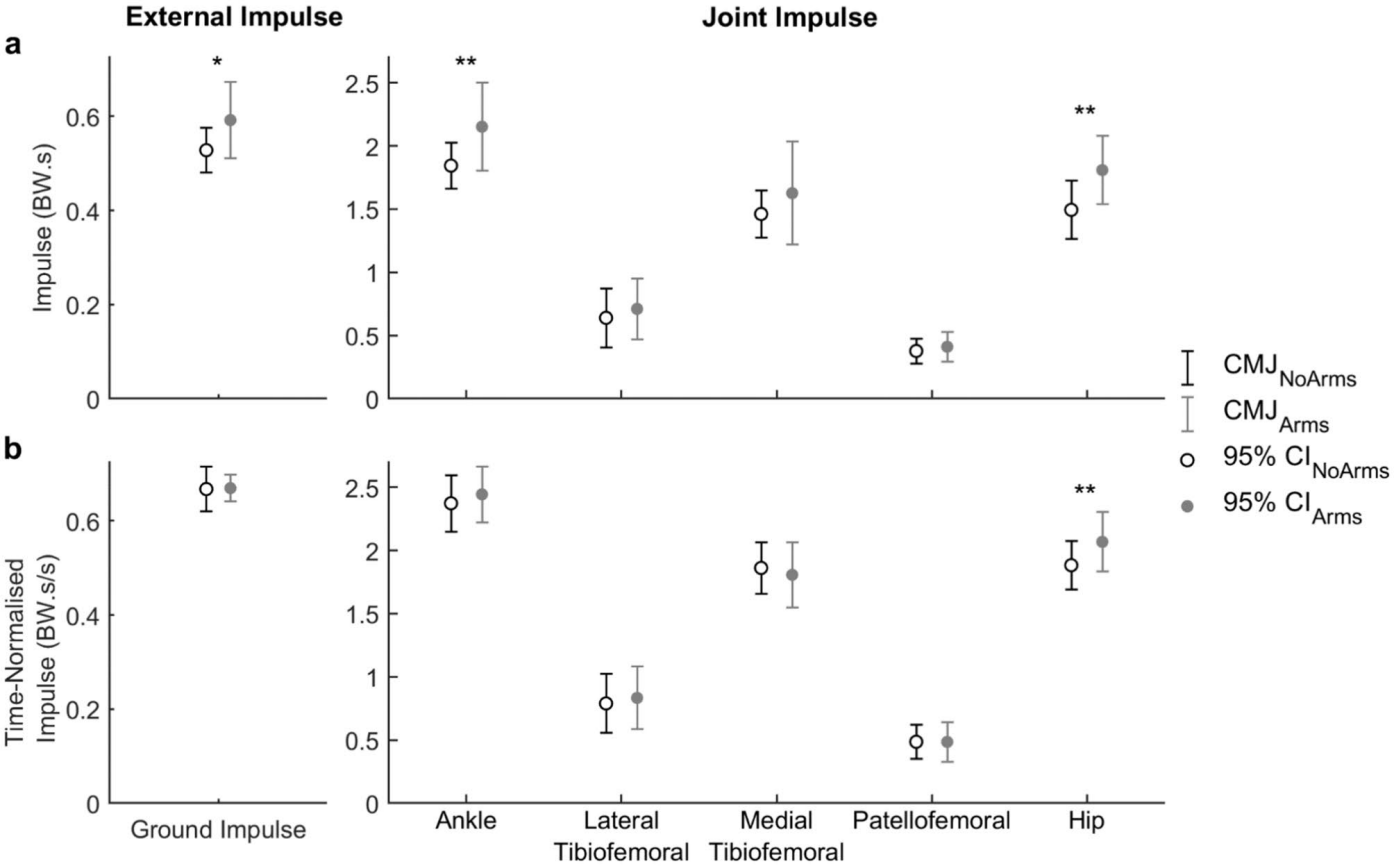
(**a**) Mean external vertical ground impulses and joint impulses for the countermovement jump without (open black markers) and with (solid grey markers) the use of an arm swing. (**b**) Time-normalised mean external vertical ground impulses and joint impulses for the countermovement jump without (open black markers) and with (solid grey markers) the use of an arm swing. The capped lines show the upper and lower bounds of the Bonferroni adjusted 95% confidence interval (CI) of the mean impulses for CMJ_NoArms_ (black line) and CMJ_Arms_ (grey line). Significant differences between CMJ_NoArms_ and CMJ_Arms_ are noted with single (*, *p* < 0.05) or double (**, *p* < 0.01) asterisks.

Significant differences in external impulse were found between CMJ_NoArms_ and CMJ_Arms_ (*p* = 0.012; medians: CMJ_NoArms_ = 0.515 BW·s, CMJ_Arms_ = 0.571 BW·s). There was also a significant main effect of the use of arm swing on vertical joint impulse (*p* = 0.009; mean difference of 0.178 BW·s, 95% CI, 0.051 to 0.305 BW·s) (Fig. 1a). However, a significant simple effect of the use of an arm swing on the vertical joint impulses was only found at the ankle (*p* = 0.009, mean difference = 0.307 BW·s) and hip joints (*p* < 0.001, mean difference = 0.315 BW·s) (Fig. 1a). When normalising for time, the external vertical impulse was similar for both the CMJ_NoArms_ (0.669 BW·s·s^−1^) and CMJ_Arms_ (0.667 BW·s·s^−1^). The main effect of an arm swing on vertical joint impulse (*p* = 0.158) and simple effect of the use of an arm swing on vertical joint impulses at the ankle (*p* = 0.324) were no longer statistically significant (Fig. 1b). The simple effect of an arm swing on time-normalised vertical joint impulse remained significantly different only at the hip joint (*p* = 0.006, mean difference = 0.185 BW·s·s^−1^).

### Participant-level PCA

Participants exhibited between one and three fDOFs during the CMJ_NoArms_ and CMJ_Arms_ (Table 3). The main effect of the use of arm swing on cumulative explained variation of the muscle forces and 3D JCFs by PC1 to PC3 was significant (*p* = 0.034, mean difference = 0.975%) (Table 3). This resulted in a higher number of fDOF present during the CMJ_Arms_ compared to CMJ_NoArms_. There was also a weak trend in which participants who exhibited 2 fDOF had a higher external vertical impulse than those with only 1 fDOF, and a similar or higher impulse than those requiring 3 fDOF (Table 3).

**Table 3:** (a) Number of PCs required to explain 95% of the 39 composite muscle forces (original DOF in input matrix: 501 data points × 39 participant composite muscles) and 15 joint contact forces (original DOF in input matrix: 501 data points × 15 forces (5 joins × 3 dimensions)) per participant (n = 18) and the average external vertical impulse (mean ± standard deviation) grouped by number of PCs required for CMJ_NoArms_ and CMJ_Arms_. (b) Mean ± standard deviation of the cumulative explained percentage of the muscle forces and joint contact forces by PC1, PC2 and PC3 for CMJ_NoArms_ and CMJ_Arms_.

	Muscle forces					Joint contact forces				
a)	Original DOF	Mean fDOF	No. of fDOF	Freq	Mean external vertical impulse (BW·s)	Original DOF	Mean fDOF	No. of fDOF	Freq	Mean external vertical impulse (BW·s)
CMJ_NoArms_	39	1.78	1	4	0.52 ± 0.04	15	2	1	2	0.55 ± 0.03
			2	14	0.61 ± 0.17			2	14	0.61 ± 0.18
			3	0	n/a			3	2	0.52 ± 0.08
CMJ_Arms_	39	2	1	1	0.51	15	2.28	1	0	n/a
			2	16	0.51 ± 0.07			2	13	0.53 ± 0.06
			3	1	0.81			3	5	0.53 ± 0.16

b)	PC1 (%)		PC2 (%)		PC3 (%)	PC1 (%)		PC2 (%)		PC3 (%)
CMJ_NoArms_^a^	83.4 ± 7.9		98.1 ± 0.9		99.1 ± 0.5	89.8 ± 4.5		97.0 ± 1.5		98.8 ± 0.6
CMJ_Arms_^a^	85.5 ± 8.3		97.2 ± 1.3		98.9 ± 0.5	87.6 ± 4.9		96.0 ± 2.0		98.2 ± 0.9
Significance	0.606		0.007^b^		0.014^b^	0.007^b^		0.015^b^		0.002^b^

^a^There was a significant main effect for the use of an arm swing in the percentage of cumulative explained variation, mean difference = 0.975%, *p* = .034.

^b^There was a significant difference (*p* < .017—Bonferroni adjustment for 3 PC levels) for the cumulative explained variation between CMJ_NoArms_ and CMJ_Arms_.

### Group-level PCA

**Figure 2:**
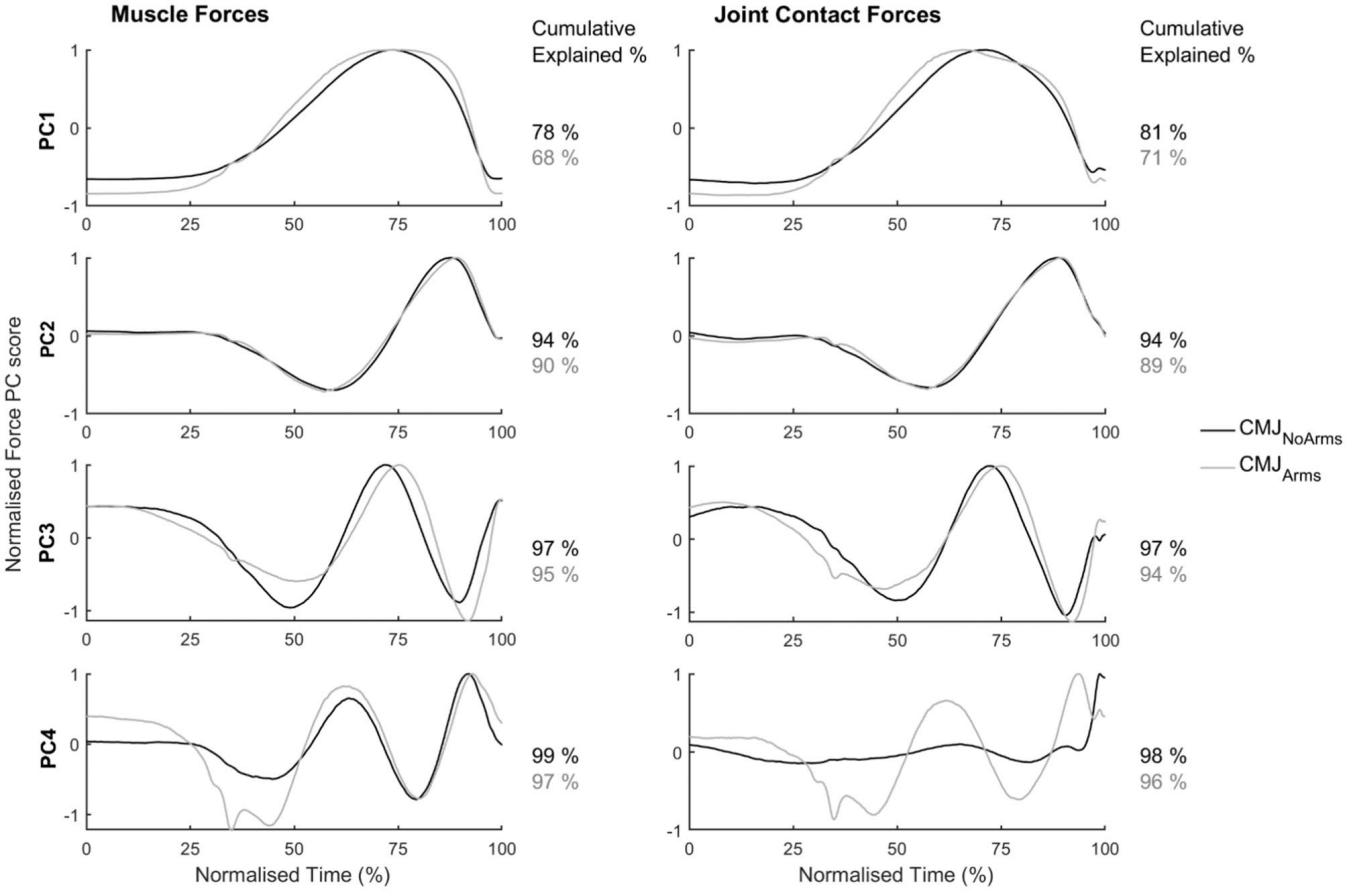
Normalised principal component score curves and their respective cumulative explained variation (%) describing the muscle forces (left) and 3-dimensional joint contact forces (right) for a countermovement jump without (CMJ_NoArms_, black lines) and with (CMJ_Arms_, grey lines) the use of an arm swing. The muscle force’s PC3 CMJ_Arms_ and joint contact force’s PC1 were inverted for comparison.

Figure 2 shows the first four normalised PC curves describing the group’s muscle and 3D JCFs for the CMJ_NoArms_ and CMJ_Arms_. Muscle force PC3 for CMJ_Arms_ and joint contact force PC1 for both jumps were inverted in Fig. 2 for comparison. An increase in frequency can be seen from PC1 to PC4, with the maxima and minima, particularly in PC3, being delayed for CMJ_Arms_ (peak 1 = 50%, peak 2 = 75.2%, peak 3 = 91.6%) compared to CMJ_NoArms_ (peak 1 = 48.8%, peak 2 = 71.7%, peak 3 = 89.8%). A higher percentage of cumulative variation is explained for both variables for the CMJ_NoArms_ (e.g. PC1—3: muscles = 97.3%; 3D JCFs: 96.6%) than with the same number of PCs for the CMJ_Arms_ (e.g. PC1—3: muscles = 95.1%; 3D JCFs: 93.3%). The 270 DOF present for the group’s 3D JCF were described by 3 fDOF for CMJ_NoArms_ and 4 fDOF for CMJ_Arms_.

### Muscle groupings and their linear PC combination

**Figure 3:**
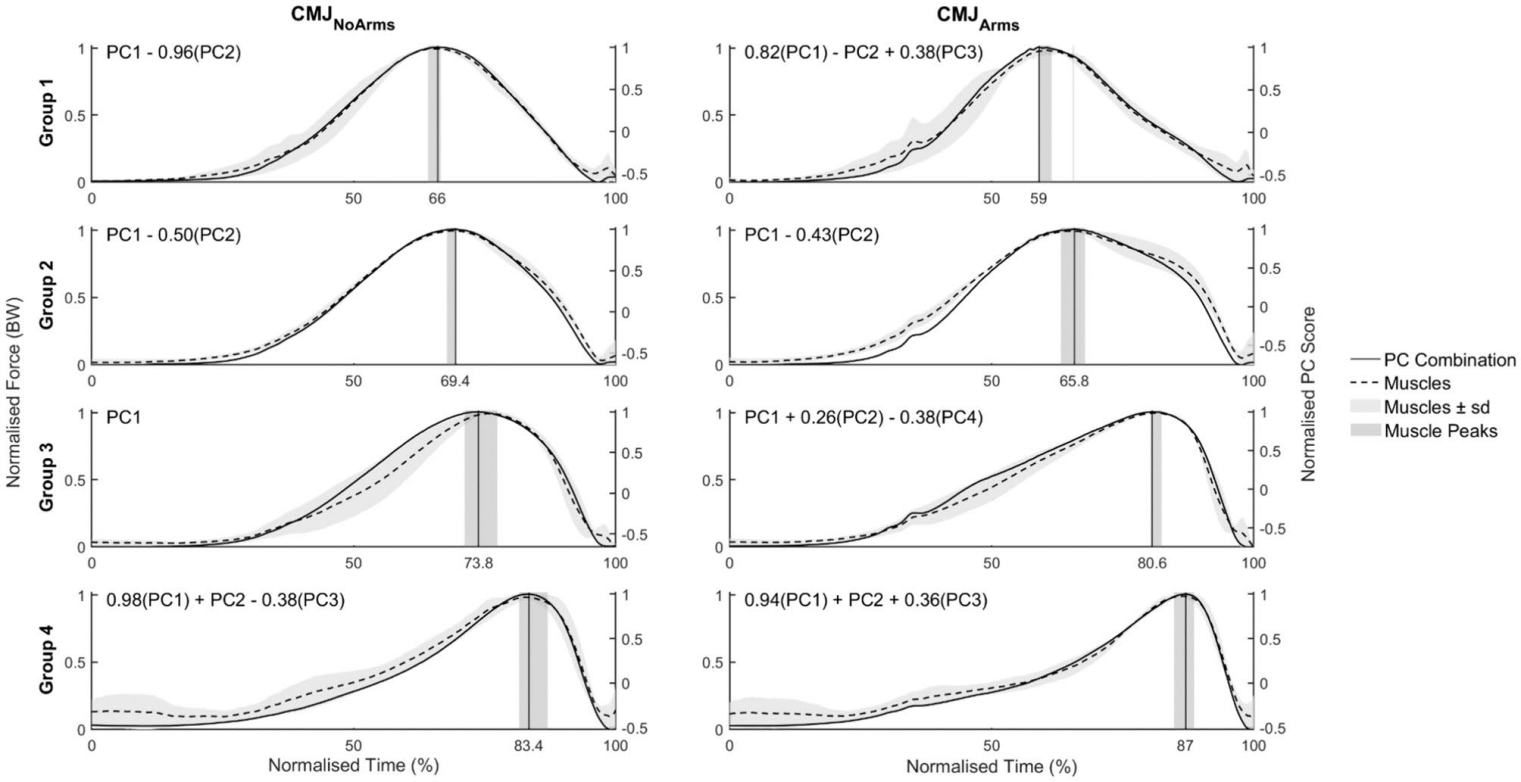
Group-level mean composite muscle force curves (dashed lines, ± 1 standard deviation (light grey shaded curve)) together with the linear composition of principal components (PC—solid line) defining the muscle group for muscle groups 1 to 4. The linear composition of principal components is defined by the equation in the top left corner of each graph. The peak of the principal component composition (vertical line) occurs within the range of peak relative timings of the group of muscles (dark grey shaded area) for the CMJ_NoArms_ (left) and CMJ_Arms_ (right).

Figure 3 depicts the mean group-composite muscle force curves for CMJ_NoArms_ and CMJ_Arms_ with their representative PC combination for muscle groups 1 to 4. These muscle groups contain the prime movers, including the hamstrings and hip stabilizers in group 1 and the gluteus maximus and medial quadriceps in group 2. Group 3 consists of the vastus lateralis and ankle stabilizers, and the soleus is included in group 4, together with the rectus femoris. The four muscle groups have sequential peak force timings. The original 702 muscle force DOFs can be described by three and four fDOF for the prime movers during the CMJ_NoArms_ and CMJ_Arms_ respectively. The subtraction of PC2 from PC1 moves the peak earlier in the countermovement (groups 1 and 2). The addition of PC3 in CMJ_Arms_ group 1 resulted in a superimposed curve at 80–90% of the countermovement. While CMJ_NoArms_ group 3 is solely defined by PC1, CMJ_Arms_ required the subtraction of PC4 to increase the rate of force development till 44% of the countermovement, which was reduced by PC2, while both PCs delay the peak defined by PC1. In group 4, the peak was delayed by the subtraction or addition of PC3 for CMJ_NoArms_ and CMJ_Arms_ respectively due to their opposing profiles. While the stabilising muscles in groups 5 to 7 are well defined for the CMJ_NoArms_, only group 5’s PC combination vaguely followed the muscle profiles for CMJ_Arms_. The mean group-composite muscle force curves with their representative PC combination can be found in Supplementary Fig. S1 online.

### Real time comparison of CMJ_NoArms_ and CMJ_Arms_

**Figure 4:**
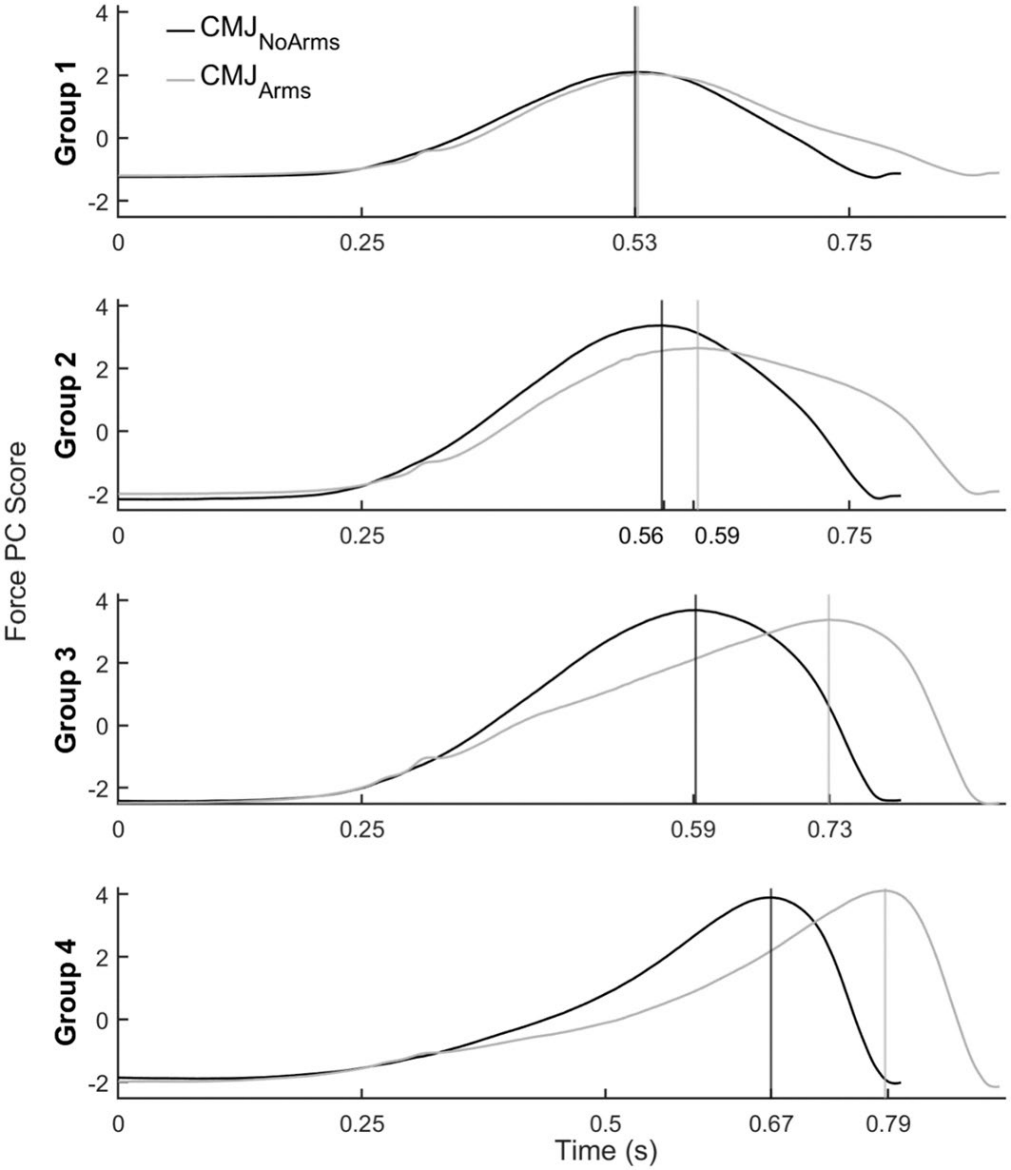
Linear combinations of PC score curves defining four muscle groups plotted against the average length of the CMJ_NoArms_ (0.803 s, black line) and CMJ_Arms_ (0.904 s, grey line) in order of peak timings (vertical lines).

The real time differences between the CMJ_Arms_ (0.904 s) and CMJ_NoArms_ (0.803 s) curves can be seen in Fig. 4. The delay between the peak force of group 1’s hip extensor and group 3’s knee extensor is prolonged in CMJ_Arms_ (0.196 s) compared to CMJ_NoArms_ (0.062 s). However, the peaks of muscle group 1 (CMJ_NoArms_ = 0.530 s, CMJ_Arms_ = 0.533 s) and group 2 (CMJ_NoArms_ = 0.557 s, CMJ_Arms_ = 0.595 s) occur at similar times for both jumps. The time delay between the peaks of the knee extensor in group 3 to the plantar flexor in group 4 (CMJ_NoArms_ = 0.077 s, CMJ_Arms_ = 0.058 s) and plantar flexor in group 4 to take-off (CMJ_NoArms_ = 0.133 s, CMJ_Arms_ = 0.117 s) is also similar between jumps. There was a statistically significant, strong positive correlation between principal impulse and muscle impulse (r(8) = 0.994, *p* < 0.001).

## Discussion

The purpose of this study was to understand the effect of an arm swing during a CMJ on performance and fDOF. The results confirmed the hypothesis of improved performance with the use of an arm swing during a CMJ through increased external and joint impulses, particularly at the hip and ankle joints, and an increased joint extension proximal-to-distal delay. This study showed that the key characteristics of the movement which had 270 kinetic DOFs and 702 muscle force DOFs at a group level, could be described by a reduced number of fDOF. The CMJ_Arms_ exhibited four fDOF to define muscle forces and 3D JCFs at a group level, while the CMJ_NoArms_ resulted in only three fDOF for both variables. This confirms the second hypothesis that more fDOF are utilized in CMJ_Arms_ compared to CMJ_NoArms_.

The prolonged countermovement due to the use of an arm swing resulted in increased vertical external impulse, in agreement with previous literature^16–19^, and increased hip and ankle joint impulses, which is in agreement with Chiu et al.^20^ and Hara et al.^21^ who found a similar pattern in the NJM. However, it was only the hip that exhibited an improved vertical JCF-time profile with an added arm swing, as it was the only joint impulse that remained significantly greater after removing the effect of increased time (by time normalization). This can be explained by the increase in delay in the proximal-to-distal strategy which occurred between peak hip and knee extensor forces, but not between the knee extensors, plantar flexors and take-off. The time between hip and knee extensor peaks increased to allow the forward arm swing to begin the upward acceleration and propulsive phase before the leg begins to extend^17^, maximising energy transfer from the elbows and shoulders to the trunk and pelvis creating the ‘pull mechanism’^18^. This resulted in slower hip extension and muscle contraction within an improved region of the force–velocity curve at which the muscles are able to generate more force^28^. Therefore, the inclusion of an arm swing likely improves the hip JCF-time profile by enhancing the proximal-to-distal strategy of the countermovement.

Unlike the increase in time-normalised impulse at the hip, the vertical external and ankle impulses only increased when calculated in real time, indicating that a longer ground contact time was the main factor contributing to increased impulse. However, previous studies have shown an increase in ankle NJM with the use of an arm swing in a CMJ^16–18,20,21^. Even though the average vertical JCF did not increase significantly, the ankle NJM may have increased due to the larger moment arm created about the ankle joint due to the anterior projection of the centre of mass with an arm swing^20^.

The vertical impulses at the tibiofemoral and patellofemoral joints did not increase with an added arm swing, even when time was not normalised. As previously discussed, the delay in the proximal-to-distal strategy with the added arm swing occurs just after the breaking phase of the countermovement and prior to leg extension, increasing the forces produced by the biarticulate hamstrings and the gastrocnemius. These muscles generate a flexion moment on the tibia and femur respectively, requiring the knee extensors to be used maximally during the closed chain extension present in jumping^10^. The patellofemoral JCF has been modelled based on the quadriceps tendon and patellar tendon forces while the tibiofemoral JCF was modelled as a result of the opposing posterior cruciate ligament and patellar tendon forces^29^. The maximally used quadriceps during the CMJ_NoArms_ and CMJ_Arms_, together with their diminished ability to transfer tension to the patellar tendon while in knee flexion^9^ (the posture which is prolonged during CMJ_Arms_), may explain the similar tibiofemoral and patellofemoral vertical impulses during both jumps.

The results of the participant-level PCA where only one to three fDOF were required to describe the main characteristics of both the CMJ_NoArms_ and CMJ_Arms_, suggests that a large number of constraints exist in individual CMJ motor control strategies. On average, only one additional fDOF was needed to define the 3D JCF and muscle forces separately for all 18 participants in the CMJ_NoArms_ (three total fDOF) and two additional fDOF were needed for CMJ_Arms_ (four total fDOF). This shows that there was a high degree of similarity between participants in their proximal-to-distal movement pattern, suggesting that underlying mechanical constraints exist within our musculoskeletal system, enforcing this movement pattern^13^. At the maximum depth of the countermovement before the propulsion phase, the quadriceps are able to generate more force about the femur through the quadriceps tendon than about the tibia through the patellar tendon due to the knee flexion and geometry of patella^9^. This enhances the proximal-to-distal strategy in which the femur extends prior to the tibia in vertical jumping^29^. The biarticulate muscles, apart from creating a rotational effect not only on their proximal and distal segments, but also on their intermediate segment^30^, are able to transfer energy from their proximal-to-distal segment^10^. Therefore, the biarticulate muscles and the geometry of the patella define additional constraints on the system through mechanical coupling during vertical jumps, reinforcing the proximal-to-distal delay and reducing the load on the central control system^8^.

While the lower limb segments exhibit mechanical coupling, the anatomy of the upper limb is not mechanically linked and constrained to that of the lower limb. Thus, the inclusion of an arm swing exhibited greater variability both within (participant-level fDOF) and between (group-level fDOF) participants' movement strategies. Theoretically, simply prolonging the proximal-to-distal strategy in CMJ_Arms_ could have been defined by only three fDOF at a group level, particularly as the peaks in PC2 and PC3 are already delayed in CMJ_Arms_ compared to CMJ_NoArms_. However, CMJ_Arms_ required the inclusion of PC4 to describe the force–time curve of the vastus lateralis as some participants exhibited double curves or multiple peaks in the knee extensor force–time curve, while others exhibited a smoother single curve. Similar patterns have been found in knee extensor NJM, where a smoother curve resulted in improved jump performance, compared to knee NJMs consisting of multiple peaks^20^. The CMJ_Arms_ also required an additional use of PC3 in muscle group 1 (including the biceps femoris), which describes a slight increase in muscle force late in the CMJ propulsion phase. This may have been caused by the delayed peak of the knee extensor requiring additional antagonistic co-contraction of the biarticulate biceps femoris for stability at the knee joint to avoid hyperextension on take-off^8,9^.

The effect of increased variability and number of fDOF on jump performance can be seen more clearly on a participant level. Participants exhibiting two fDOF had a higher vertical external impulse compared to those who only had one fDOF (Table 3) as it would represent the majority of muscles working simultaneously. At a participant level, two fDOF are sufficient to define a proximal-to-distal strategy as the PC score curves are able to follow the individual’s strategy more closely than at a group level and reduces the need for more generic PC curves. The addition of the third fDOF at participant level may indicate excessive variation and reduced coordination within the individual’s motor strategy, resulting in decreased performance. However, the sample size of participants exhibiting one and three fDOFs is too small to make conclusive comparisons between the groups.

The addition of an arm swing increased the complexity of the movement, resulting in more variation of the lower limb motor strategies being explored to achieve maximum height in the CMJ. This occurred as the arms are not directly mechanically coupled to the lower extremity, and therefore resulted in an additional independent fDOF present in CMJ_Arms_ compared to CMJ_NoArms_. Other possible sources of variation in CMJ_Arms_ which may affect and improve jump height include arm swing timing and technique^17,19^. Individual’s dynamic core flexion strength has been shown to affect the musculoskeletal ability to transfer the energy generated from the arms to the distal segments^31^. Due to the contribution of shoulder musculature to the vertical energy generated by the arm swing, it has been suggested that shoulder flexor strength may also alter performance in CMJ_Arms_^22^, although further research is needed. Through training, it may be possible to reduce the variation within the lower limb movement strategies. This would reduce the independent fDOF present, therefore reducing burden on the motor control system. Achieving maximum height in a jump with the use of an arm swing is important in various sports, such as basketball, volleyball and handball. However, additional unexpected demands and constraints are inherent within the sports. The reduction of the fDOF in maximal CMJ_Arms_ may increase the capacity of the central control system to adapt to these unexpected sport demands. Therefore, training a specific optimal technique, and increasing shoulder and dynamic core flexion strength may be crucial to improve jump and sport performance.

As noted by Cleather and Cushion^32^, even though the motor strategies used in both CMJs can be described by three or four fDOF, it does not imply that every participant’s motor strategy is the same. In fact, the PC combination for each participant and each muscle can be easily identified from the PCA’s coefficient matrix, resulting in different curve profiles, peak timings and motor strategies from the same PCs. The small number of fDOF present simply indicate that the muscle forces and JCFs are tightly constrained during CMJs and that individual strategies can be defined by a linear combination of the same PCs^32^, as can be seen from the multiple curves resulting from different combinations of the CMJ’s PCs (Fig. 3). Future research may explore whether additional constraints are present between the lower extremity, trunk and upper limb segments through a full-body musculoskeletal model and PCA. Jump performance can also be investigated to identify the movement characteristics describing the most effective arm swing technique to improve jump height.

A strength of this study is the inclusion of time normalisation in the vertical impulse analysis. This allowed for a distinction between solely the difference in time or a combined difference of time and vertical force as the main contribution for change in impulse between CMJ_NoArms_ and CMJ_Arms_. Another strength is the comparison of the muscle group principal component combinations between jumps in real time, clearly indicating the similarities and differences in the timings of the proximal-to-distal strategy. This detail is lost when comparing muscle activity in normalised time^17^. Although Kovács et al.^17^ found the same sequence of muscle peak timings using electromyography, no differences were found in muscle peak timings between the jumps. This can be misleading as true differences in peak timing may still exist due to the prolonged CMJ_Arms_, while the difference found in the vastus lateralis activation between 38 and 56% of CMJ_Arms_ and CMJ_NoArms_ may not be significant in real time. The principal impulse for each muscle group is also significantly correlated to the sum of the group’s muscle impulses, with a strong positive correlation. This demonstrates further the usefulness of PCA as a data reduction method to simplify the understanding of complex motor control strategies.

However, it is important to keep in mind the limitations of this study. Firstly, only the right side of the athlete was analysed in this study. Secondly, the models employed were linearly scaled for each participant rather than using participant-specific models. However, the aim of this study was to compare the performance and fDOF of a CMJ with and without the use of an arm swing. Since a linearly scaled model of the right lower limb was used for all participants and both the CMJ_NoArms_ and CMJ_Arms_, these limitations should not affect our comparison results. Additionally, it must be noted that the PCA technique is based solely on linear relationships between the DOF. Therefore, the number of fDOF may be overestimated as non-linear relationships may still exist in the identified fDOF and thus may not be entirely independent^1^. Even though a rigorous method was followed for these steps, muscle group categorization and cut-off coefficients for the group’s PC combination were determined using mainly a qualitative visual inspection of the curves. Finally, individual strategies may have varied and slightly different muscle group categorizations and PC linear combinations may have emerged between participants. There were several possible ways to interpret the results of this study, however the method followed was chosen due to the similarity in movement strategies found previously between participants for CMJ_Arms_ and CMJ_NoArms_^13^, suggesting that an in-depth group analysis was suitable to compare muscle forces between jumps.

In conclusion, jump performance improved with an added arm swing as it increased the ground contact time, resulting in higher vertical impulses. The increased ground contact time to perform the arm swing was mainly used by the lower extremity to decrease the hip extension velocity, allowing the hip muscles to generate higher forces, and to delay knee extension, enhancing the proximal-to-distal strategy. The PCA has shown that muscle activation and joint kinetics in the lower limb exhibit very similar patterns within and between individuals. This suggests that the underlying anatomy, such as biarticulate muscles and the patella, provide mechanical constraints and coupling during a CMJ, reducing the load on the central control system^8^. The inclusion of an arm swing required an additional fDOF (four in total) to describe the main characteristics of the movement, suggesting that the arms are not directly mechanically coupled with the lower extremity, resulting in additional variation within individual motor strategies.

### Supplementary Information

Supplementary Information.

## Data Availability

The datasets collected by Cushion et al.^13^ which were analysed during this study are available from the corresponding author on reasonable request.
