# Supplementary material for: An arm swing enhances the proximal-to-distal delay in joint extension during a countermovement jump
Source: Sci Rep. 2024 Sep 2;14:20371. doi: 10.1038/s41598-024-70194-z (PMC11368957; doi:10.1038/s41598-024-70194-z)
Supplement: Supplementary file 1 — Supplementary Information. [file 41598_2024_70194_MOESM1_ESM.pdf]

Ms Christina M. Cefai, Dr Joseph W. Shaw, Dr Emily J. Cushion, and Dr Daniel J. Cleather

An arm swing enhances the proximal-to-distal delay in joint extension during a countermovement jump.

Supplementary Results

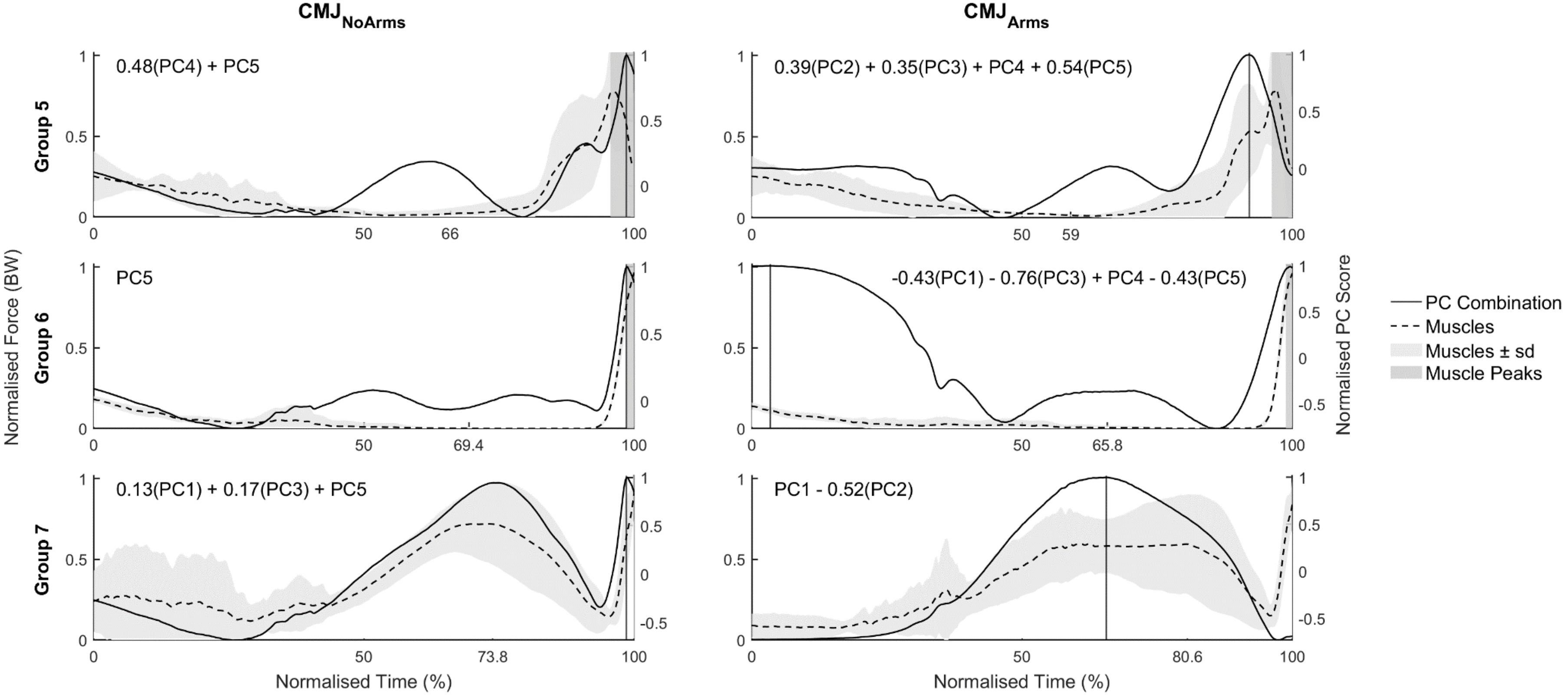

Supplementary Fig. S1. Group-level mean composite muscle force curves (dashed lines, ± 1 standard deviation (light grey shaded curve)) and force profile together with the linear composition of principal components (PC - solid line) defining the muscle group for muscle groups 5 to 7. The linear composition of principal components are defined by the equation in the top left corner of each graph. The peak of the principal component composition (vertical line) occurs within the range of peak relative timings of the group of muscles (dark grey shaded area) for the CMJ_{NoArms} (left) and CMJ_{Arms} (right).
